# Supplementary material for: Self-generated morphology in lagoon reefs
Source: PeerJ. 2015 May 12;3:e935. doi: 10.7717/peerj.935 (PMC4499466; doi:10.7717/peerj.935)
Supplement: Data S2 [file peerj-03-935-s002.docx]

# Turbo Pascal code for the basic reef growth model. See page 7 for modifications to the procedure ‘applyrules’ that implement the collapse limit. This code and the concepts within are freely available for use and modification, with acknowledgement. As Turbo Pascal is now obsolete we are migrating to a new language. To join the distribution list for the updated model please email fathom5marineresearch@gmail.com with subject line ‘reef growth model’.

program reef6;

const

maxsize = 250; {size of data array}

size = 160; {size of recruitment array centred within data array}

type

site = record

depth: integer;

life: boolean;

end;

bigarray = array[1..maxsize, 1..maxsize] of site; {defines array to store boundary coordinates}

bigptr = ^bigarray;

bighandle = ^bigptr;

var

infile, outfile: text;

i, j, numberecruits, maxdepth, numberloops, savenumber: longint;

startx, starty, minneighbours: longint;

infilename, outfilename: str255;

probH: real;

datetime: datetimerec;

rec: rect;

bhcurrent, bhfuture: bighandle;

ran0y: extended;

ran0v: array[1..97] of extended;

seed: longint;

key: char;

procedure makearray;

begin

bhcurrent := bighandle(newhandle(sizeof(bigarray)));

if bhcurrent = nil then

begin

sysbeep(3);

showtext;

writeln('***error, could not create large data array in heap***');

exittoshell;

end;

Hlock(handle(bhcurrent));

bhfuture := bighandle(newhandle(sizeof(bigarray)));

if bhfuture = nil then

begin

sysbeep(3);

showtext;

writeln('***error, could not create large data array in heap***');

exittoshell;

end;

Hlock(handle(bhfuture));

end;

procedure initarray;

var

m, n: longint;

begin

for m := 1 to maxsize do

for n := 1 to maxsize do

begin

bhcurrent^^[m, n].depth := maxdepth;

end;

for m := 1 to maxsize do

for n := 1 to maxsize do

begin

bhfuture^^[m, n].depth := maxdepth;

end;

end;

procedure writeoutput;

var

i, j: integer;

begin

writeln;

outfilename := newfilename('save data as ?');

rewrite(outfile, outfilename);

for i := 1 to maxsize do

begin

for j := 1 to maxsize - 1 do

write(outfile, bhcurrent^^[i, j].depth : 2, ' ');

writeln(outfile, bhcurrent^^[maxsize, j].depth : 2);

end;

close(outfile);

end;

function ran0 (var idum: longint): extended; {calculates random numbers}

var

dum: extended;

j: longint;

begin

if idum < 0 then

begin

randseed := -idum;

idum := 1;

for j := 1 to 97 do

dum := abs(random / 32768);

for j := 1 to 97 do

ran0v[j] := abs(random / 32768);

ran0y := abs(random / 32768);

end;

j := 1 + trunc(96.0 * ran0y);

if (j > 97) or (j < 1) then

begin

writeln('pause in routine ran0');

readln

end;

ran0y := ran0v[j];

ran0 := ran0y;

ran0v[j] := abs(random) / 32768;

end;

procedure readtext; {reads values from a tab delimited text file}

var

i, j, n, k, value, rowlength: longint;

begin

infilename := oldfilename('open coord. file');

reset(infile, infilename);

for j := maxsize downto 1 do

for i := 1 to maxsize do

begin

read(infile, value);

{writeln('i= ', i : 2, ' j= ', j : 2, ' = ', value : 4);}

if value <= maxdepth then

bhcurrent^^[i, j].depth := value;

if value > maxdepth then

begin

sysbeep(3);

showtext;

writeln;

writeln('***error, seafloor depth exceeded***');

exittoshell;

end;

end;

close(infile);

end;

procedure init;

begin

writeln('This program was written by Michael Hamblin, UWA, 29-4-96');

writeln('It models reef growth in x-y and z from randomly spaced coral recruits');

writeln('the dimensions of the initial seafloor grid in metres are: ', maxsize : 3, ' by ', maxsize : 3);

writeln('the dimensions of the initial recruitment grid in metres are: ', size : 3, ' by ', size : 3);

writeln;

writeln('enter seafloor depth in metres');

readln(maxdepth);

writeln;

writeln('to input data from a file enter "i", to begin a new reef enter any other key');

readln(key);

if key = 'i' then

readtext

else

begin

writeln;

writeln('enter integer number of initial recruits');

readln(numberecruits);

end;

writeln;

writeln('enter minimum number of live neighbours to guarantee growth, 1-17');

readln(minneighbours);

if (minneighbours < 1) or (minneighbours > 17) then

begin

writeln;

writeln('***error, number must be between 1 and 17 inclusive***');

writeln;

writeln('enter minimum number of live neighbours to guarantee growth, 1-17');

readln(minneighbours);

end;

writeln;

writeln('enter iteration multiples to save data');

readln(savenumber);

writeln;

writeln('enter total number of growth iterations');

readln(numberloops);

gettime(datetime);

seed := -1 * datetime.second;

startx := trunc((maxsize - size) / 2) + 1;

starty := trunc((maxsize - size) / 2) + 1;

end;

procedure recruit;

var

x, y: real;

i, j, k: integer;

begin

k := 0;

repeat

x := ran0(seed);

y := ran0(seed);

if (x < 0) or (x > 1) then

writeln('***error , ran0 exceeded 0 - 1*** x=', x : 10 : 10);

if (y < 0) or (y > 1) then

writeln('***error , ran0 exceeded 0 - 1*** y=', y : 10 : 10);

{writeln('x= ', x : 4 : 4, ' y= ', y : 4 : 4);}

i := trunc(x * (size) + startx);

j := trunc(y * (size) + starty);

{writeln('i= ', i : 4, ' j= ', j : 4);}

bhcurrent^^[i, j].depth := bhcurrent^^[i, j].depth - 1;

k := k + 1;

until k = numberecruits;

end;

procedure applyrules;

var

isolated: boolean;

currentdepth, i, j: integer;

probH, probV, x, y, z, countH: real;

begin

for i := 1 to maxsize do

for j := 1 to maxsize do

begin

bhfuture^^[i, j].depth := bhcurrent^^[i, j].depth;

end;

for i := 2 to maxsize - 1 do

for j := 2 to maxsize - 1 do

begin

countH := 0;

currentdepth := bhcurrent^^[i, j].depth;

if (currentdepth > 0) then {sum neighbours within eight cell neighbourhood – max. 16}

begin

if (currentdepth - bhcurrent^^[i - 1, j - 1].depth = 1) then

countH := countH + 1;

if (currentdepth - bhcurrent^^[i - 1, j - 1].depth > 1) then

countH := countH + 2;

if (currentdepth - bhcurrent^^[i - 1, j].depth = 1) then

countH := countH + 1;

if (currentdepth - bhcurrent^^[i - 1, j].depth > 1) then

countH := countH + 2;

if (currentdepth - bhcurrent^^[i - 1, j + 1].depth = 1) then

countH := countH + 1;

if (currentdepth - bhcurrent^^[i - 1, j + 1].depth > 1) then

countH := countH + 2;

if (currentdepth - bhcurrent^^[i, j + 1].depth = 1) then

countH := countH + 1;

if (currentdepth - bhcurrent^^[i, j + 1].depth > 1) then

countH := countH + 2;

if (currentdepth - bhcurrent^^[i + 1, j + 1].depth = 1) then

countH := countH + 1;

if (currentdepth - bhcurrent^^[i + 1, j + 1].depth > 1) then

countH := countH + 2;

if (currentdepth - bhcurrent^^[i + 1, j].depth = 1) then

countH := countH + 1;

if (currentdepth - bhcurrent^^[i + 1, j].depth > 1) then

countH := countH + 2;

if (currentdepth - bhcurrent^^[i + 1, j - 1].depth = 1) then

countH := countH + 1;

if (currentdepth - bhcurrent^^[i + 1, j - 1].depth > 1) then

countH := countH + 2;

if (currentdepth - bhcurrent^^[i, j - 1].depth = 1) then

countH := countH + 1;

if (currentdepth - bhcurrent^^[i, j - 1].depth > 1) then

countH := countH + 2;

x := ran0(seed);

y := ran0(seed);

if countH >= minneighbours then

ProbH := 1;

if countH < minneighbours then

probH := x * countH * 0.0625;

if (probH > y) then {the coral is overgrown by a neighbour}

bhfuture^^[i, j].depth := bhcurrent^^[i, j].depth - 1;

end;

if (currentdepth > 0) and (currentdepth < maxdepth) then

begin {assign a random vertical growth probability}

probV := ran0(seed);

z := ran0(seed);

if (probV < 0) or (probV > 1) or (x < 0) or (x > 1) then

writeln('* * Error , ran0 exceeded 0 - 1 * * x=', x : 10 : 10);

if (probV > z) then {the coral grows vertically}

bhfuture^^[i, j].depth := bhcurrent^^[i, j].depth - 1;

end;

end; {end for loop to search grid}

for i := 1 to maxsize do

for j := 1 to maxsize do

begin

bhcurrent^^[i, j].depth := bhfuture^^[i, j].depth;

end;

end;

begin

hideall;

setrect(rec, 0, 40, 550, 450);

settextrect(rec);

showtext;

makearray;

init;

if key <> 'i' then

begin

initarray;

recruit;

writeln('save initial recruits');

writeoutput; {to display on screen as text change procedure "writeoutput" to "writetext"}

end;

for i := 1 to numberloops do

begin

applyrules;

writeln(i : 2, ' loops completed');

if i mod savenumber = 0 then

writeoutput; {to display on screen as text change procedure "writeoutput" to "writetext"}

end;

writeln;

writeln('iterations= ', numberloops : 2);

Hunlock(handle(bhcurrent));

disposhandle(handle(bhcurrent));

Hunlock(handle(bhfuture));

disposhandle(handle(bhfuture));

end.

Modifications to the procedure ‘applyrules’ that implement the collapse limit. This procedure calculates the height of each cell above each of its neighbours, and prevents the cells from growing if this value equals or exceeds the predefined ‘collapselimit’ (in this case 2m).

program reef6;

const

maxsize = 250; {size of data array}

size = 160; {size of recruitment array centred within data array}

collapselimit = 2; {maximum height in metres corals can project above any neighbour}

procedure applyrules;

var

isolated: boolean;

currentdepth, i, j: integer;

probH, probV, x, y, z, countH: real;

begin

for i := 1 to maxsize do

for j := 1 to maxsize do

begin

bhfuture^^[i, j].depth := bhcurrent^^[i, j].depth;

end;

for i := 2 to maxsize - 1 do

for j := 2 to maxsize - 1 do

begin

countH := 0;

currentdepth := bhcurrent^^[i, j].depth;

isolated := false; {later set to true if corals project by the defined 'collapselimit'}

if (currentdepth > 0) then {sum neighbours within eight cell neighbourhood – max. 16}

begin

if (currentdepth - bhcurrent^^[i - 1, j - 1].depth = 1) then

countH := countH + 1;

if (currentdepth - bhcurrent^^[i - 1, j - 1].depth > 1) then

countH := countH + 2;

if bhcurrent^^[i - 1, j - 1].depth - currentdepth >= collapselimit then

isolated := true;

if (currentdepth - bhcurrent^^[i - 1, j].depth = 1) then

countH := countH + 1;

if (currentdepth - bhcurrent^^[i - 1, j].depth > 1) then

countH := countH + 2;

if bhcurrent^^[i - 1, j].depth - currentdepth >= collapselimit then

isolated := true;

if (currentdepth - bhcurrent^^[i - 1, j + 1].depth = 1) then

countH := countH + 1;

if (currentdepth - bhcurrent^^[i - 1, j + 1].depth > 1) then

countH := countH + 2;

if bhcurrent^^[i - 1, j + 1].depth - currentdepth >= collapselimit then

isolated := true;

if (currentdepth - bhcurrent^^[i, j + 1].depth = 1) then

countH := countH + 1;

if (currentdepth - bhcurrent^^[i, j + 1].depth > 1) then

countH := countH + 2;

if bhcurrent^^[i, j + 1].depth - currentdepth >= collapselimit then

isolated := true;

if (currentdepth - bhcurrent^^[i + 1, j + 1].depth = 1) then

countH := countH + 1;

if (currentdepth - bhcurrent^^[i + 1, j + 1].depth > 1) then

countH := countH + 2;

if bhcurrent^^[i + 1, j + 1].depth - currentdepth >= collapselimit then

isolated := true;

if (currentdepth - bhcurrent^^[i + 1, j].depth = 1) then

countH := countH + 1;

if (currentdepth - bhcurrent^^[i + 1, j].depth > 1) then

countH := countH + 2;

if bhcurrent^^[i + 1, j].depth - currentdepth >= collapselimit then

isolated := true;

if (currentdepth - bhcurrent^^[i + 1, j - 1].depth = 1) then

countH := countH + 1;

if (currentdepth - bhcurrent^^[i + 1, j - 1].depth > 1) then

countH := countH + 2;

if bhcurrent^^[i + 1, j - 1].depth - currentdepth >= collapselimit then

isolated := true;

if (currentdepth - bhcurrent^^[i, j - 1].depth = 1) then

countH := countH + 1;

if (currentdepth - bhcurrent^^[i, j - 1].depth > 1) then

countH := countH + 2;

if bhcurrent^^[i, j - 1].depth - currentdepth >= collapselimit then

isolated := true;

x := ran0(seed);

y := ran0(seed);

if countH >= minneighbours then

ProbH := 1;

if countH < minneighbours then

probH := x * countH * 0.0625;

if (probH > y) and (isolated = false) then {the coral is overgrown by a neighbour}

bhfuture^^[i, j].depth := bhcurrent^^[i, j].depth - 1;

end;

if (currentdepth > 0) and (currentdepth < maxdepth) then

begin {assign a random vertical growth probability}

probV := ran0(seed);

z := ran0(seed);

if (probV < 0) or (probV > 1) or (x < 0) or (x > 1) then

writeln('* * Error , ran0 exceeded 0 - 1 * * x=', x : 10 : 10);

if (probV > z) and (isolated = false) then {the coral grows vertically}

bhfuture^^[i, j].depth := bhcurrent^^[i, j].depth - 1;

end;

end; {end for loop to search grid}

for i := 1 to maxsize do

for j := 1 to maxsize do

begin

bhcurrent^^[i, j].depth := bhfuture^^[i, j].depth;

end;

end;
